# Editorial: Advancements in image processing and analysis techniques for microphysiological systems

**DOI:** 10.3389/fbioe.2026.1881039

**Published:** 2026-06-26

**Authors:** Stephanie J. Hachey, Fruzsina R. Walter, Jennifer S. Fang

**Affiliations:** 1 Department of Molecular Biology and Biochemistry, University of California, Irvine, CA, United States; 2 HUN-REN Biological Research Centre, Szeged, Hungary; 3 Department of Cell and Molecular Biology, Tulane University, New Orleans, LA, United States; 4 Department of Physiology, Tulane University, New Orleans, LA, United States

**Keywords:** artifical intelligence (AI), image analysis, microphysiological systems, multimodal data integration, new approach methodologies (NAMs), organoids, organ-on-a-chip, quantitative image analysis (QIA)

Microphysiological systems (MPS), including organ-on-chip platforms, self-assembled organoids, and three-dimensional (3D) bioprinted constructs, have rapidly evolved into increasingly sophisticated models for studying human physiology, disease mechanisms, and therapeutic responses (Ingber, 2022). By recapitulating multicellular architecture, extracellular matrix interactions, tissue-level biochemical gradients, dynamic cellular behaviors, and (in some cases) fluid flow, these systems offer a level of physiological relevance that extends beyond conventional 2D and 3D cell culture models (Low et al., 2021). Moreover, the scalability of MPS model systems unlocks the potential for rapid (semi-) high-throughput small-molecule screening studies that are not traditionally feasible in intact tissue systems. As MPS become more sophisticated and generate data of increasing complexity and volume, a secondary challenge arises regarding the rapid, rigorous, and reproducible collection, processing, and analysis of data generated by MPS model systems. Conventional methods – often developed on prototypical MPS platforms -- are often manual and therefore difficult to scale. Thus, the MPS field is increasingly recognizing the need for a new generation of automated, artificial intelligence (AI)-driven image-processing and evaluation techniques to support robust, scalable analysis of MPS data.

**FIGURE 1:**
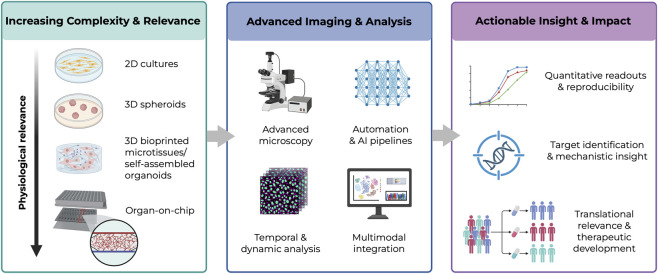
Conceptual framework for advancing image-based analysis in microphysiological systems (MPS). Increasing biological complexity and physiological relevance of MPS platforms, from conventional 2D cultures to 3D spheroids, bioprinted microtissues, self-assembled organoids, and organ-on-chip systems, generate increasingly complex and multidimensional datasets. Advanced imaging modalities, automation, artificial intelligence-driven pipelines, temporal analysis, and multimodal data integration enable the extraction of quantitative spatial, temporal, and functional information from these systems. Together, these approaches support reproducible and context-specific biological insights with applications in mechanistic studies, target identification, and translationally relevant therapeutic development. Created with Biorender.com.

This Research Topic explores next-generation image processing and analysis approaches for MPS, specifically by highlighting the transition from qualitative visualization toward integrated, computationally enabled analysis. Collectively, the contributions emphasize the importance of developing analytical frameworks capable of extracting quantitative, reproducible, and biologically meaningful information from increasingly complex systems. As illustrated in Figure 1, advances in imaging, computational analysis, and multimodal data integration across imaging, molecular, and functional readouts are evolving alongside increasingly sophisticated MPS models to enable quantitative and context-specific biological insights. But a central challenge for data collection and analysis stems from the very features that also make MPS valuable: compared with traditional monolayer cultures, 3D systems that capture the complex spatial organization and cell-cell/cell-matrix interactions of intact tissue in real time pose substantial technical challenges for image-based data collection and analysis. Current analytical frameworks adapted from conventional two-dimensional systems often fail to adequately capture the spatial organization, perfusion dynamics, signaling heterogeneity, and tissue-level interactions that define the complex MPS microenvironment (Peel and Jackman, 2020). Meanwhile, efforts to develop MPS-specific image processing approaches must consider imaging depth limitations, large multidimensional datasets, and inter-platform variability, all of which further complicate data acquisition and interpretation methods in MPS.

Several studies in this Research Topic address these limitations by developing automated, AI-driven image analysis frameworks. Yao et al. (2026) present an AI-assisted approach to quantifying melanin distribution in pigmented epidermis-on-chip models. Using a vision transformer-based segmentation framework applied to label-free brightfield imaging, the authors achieve strong concordance with expert manual annotations and enable standardized, non-invasive characterization of tissue morphology and pigmentation. Importantly, this work demonstrates that machine learning can reduce subjectivity and improve scalability in image-based analysis, facilitating longitudinal monitoring without destructive endpoint assays. Similarly, François et al. combine light sheet fluorescence microscopy (LSFM) with custom 3D computational pipelines to investigate p21 signaling dynamics after irradiation in both dispersed cells and multicellular spheroids. By reconstructing and segmenting individual nuclei within dense 3D structures, the study reveals temporal signaling behaviors that differ substantially between 2D and 3D contexts. These findings underscore that tissue architecture and extracellular matrix interactions fundamentally alter cellular responses, highlighting the importance of spatially and temporally resolved analyses for understanding complex biological behavior within MPS environments.

At the level of workflow standardization and scalability, Hachey et al. introduce an open-source image-processing pipeline for vascularized MPS. The framework enables automated quantification of tumor growth, vascular morphometry, and permeability using scalable Fiji/ImageJ-based analysis tools. By reducing manual processing time and enabling reproducible extraction of quantitative features from large imaging datasets, this work addresses a major bottleneck to wider adoption and translational implementation of vascularized MPS platforms. More broadly, it reflects a growing recognition that reproducibility and standardization in image analysis are essential for comparability across laboratories and experimental systems. Beyond imaging alone, several contributions emphasize the growing importance of multimodal, integrated analytical approaches. Truong et al. focus on reproductive and pregnancy-associated organ-on-chip systems to describe how microscopy is increasingly combined with complementary methodologies such as spatial transcriptomics, imaging cytometry, cytokine profiling, and multiplexed molecular analyses. These approaches are particularly important in complex biological systems where low cell numbers and intricate tissue organization make traditional analytical methods difficult to apply. In this context, imaging functions not as a standalone readout but as an organizing framework for integrating and interpreting molecular, spatial, and functional data.

Importantly, the studies in this Research Topic collectively challenge the notion that conventional metrics such as viability or gross morphology are sufficient to characterize advanced MPS models. As Spiller and Duarte Campos note, meaningful evaluation of 3D systems requires more comprehensive characterization of cellular state, organization, proliferation, metabolic behavior, and tissue function. This shift reflects an advancement in the field toward defining context-specific, biologically meaningful endpoints rather than relying solely on simplified surrogate measurements. As these technologies continue to expand into applications in drug development, disease modeling, and precision medicine, there is increasing emphasis on developing analytical approaches that are quantitative, reproducible, and contextually meaningful. Endpoints appropriate for mechanistic discovery studies may differ substantially from those required for therapeutic screening, toxicity assessment, or translational applications, underscoring the importance of aligning analytical strategies with the intended context of use (Alver et al., 2024).

This transition also has significant implications for the broader adoption of MPS technologies as New Approach Methodologies (NAMs) in translational and regulatory science (Zushin et al., 2023). Growing interest in alternatives to animal models has heightened the need for reproducible, scalable, and translatable datasets from human-relevant systems. In this context, automated image analysis, multimodal integration, and AI-driven quantification are increasingly important for building confidence in MPS-derived data (Moen et al., 2019). Standardized pipelines, validated performance metrics, and clinically relevant endpoints will likely play a key role in future qualification and implementation efforts (Wilkinson et al., 2016). At the same time, meaningful validation of these systems cannot occur in isolation from the clinical context (Ewart et al., 2018). Continued collaboration between engineers, computational scientists, biologists, and clinical investigators will be essential to identify which imaging-derived and multimodal features are most predictive of patient response and disease progression. Integrating patient-derived samples, co-clinical study designs, and benchmarking against clinical imaging and molecular datasets may further strengthen the translational relevance of these platforms.

Looking ahead, several priorities emerge for the field. First, base MPS model development efforts must be paired with data-driven efforts to build scalable, reproducible analytical frameworks. Resulting analytical approaches and data processing toolsets must emphasize broad platform applicability, automated data collection, physiologically relevant quantitative readouts, unbiased data integration and interpretation, and ease of use for end users. Increased effort towards these goals is essential to support broader adoption and implementation of MPS across laboratories and applications. Second, AI and machine learning approaches must continue to move beyond descriptive segmentation and towards predictive and integrative modeling. Third, image processing and analysis workflows must evolve to keep pace with increasing biological complexity, including systems that incorporate vasculature, immune components, microbiomes, and multi-organ interactions, perhaps suggesting the need for a central repository of MPS-based data analysis toolsets that can support continuous feature updates. Finally, future tools must support integration of spatial, temporal, and molecular datasets to generate a more complete understanding of tissue function and therapeutic response within complex MPS. The contributions in this Research Topic collectively reflect an emerging field of MPS data collection and analysis approaches that is already undergoing rapid evolution. In moving beyond cellular visualization alone, these studies demonstrate how advanced image processing, computational analysis, and multimodal integration are transforming MPS platforms into increasingly quantitative, reproducible, and translationally relevant tools. In doing so, they help establish the analytical foundations necessary for the next-generation of human-relevant experimental systems in biomedical research.
